# Effects of different doses of sufentanil and remifentanil combined with propofol in target-controlled infusion on stress reaction in elderly patients

**DOI:** 10.3892/etm.2013.900

**Published:** 2013-01-17

**Authors:** LI-GUO HU, JIAN-HUI PAN, JUAN LI, FANG KANG, LING JIANG

**Affiliations:** 1 Department of Anesthesiology, The Provincial Hospital Affiliated to Anhui Medical University, Hefei, Anhui 230001, P.R. China; 2 Department of Pharmacy, The Provincial Hospital Affiliated to Anhui Medical University, Hefei, Anhui 230001, P.R. China

**Keywords:** the elderly, sufentanil, remifentanil, stress, analgesia

## Abstract

The current study aimed to observe the effects of sufentanil and remifentanil combined with propofol in target-controlled infusion (TCI) on perioperative stress reaction in elderly patients. A total of 80 elderly patients requiring general anesthesia were recruited. They were divided into four groups (each n=20) according to different target concentrations of remifentanil and sufentanil. These target concentrations were: 4 ng/ml remifentanil + 0.2 ng/ml sufentanil for group I; 3 ng/ml remifentanil + 0.3 ng/ml sufentanil for group II; 2 ng/ml remifentanil + 0.5 ng/ml sufentanil for anesthesia induction and post-intubation 3 ng/ml remifentanil + 0.2 ng/ml sufentanil for anesthesia maintenance for group III; and 5 ng/ml remifentanil for anesthesia induction and post-intubation 4 ng/ml remifentanil for anesthesia maintenance for group IV. Norepinephrine (NE), epinephrine (E) and angiotensin II (Ang II) levels in plasma were measured prior to the induction of anesthesia, as well as at several different time-points following surgery. The numbers of intraoperative severe hemodynamic fluctuation, postoperative eye-opening and extubation time, and post-extubation restlessness and pain scores were recorded. Group IV had a larger circulation fluctuation control number and higher levels of NE, E and Ang II at 3 h after surgery than any other group (P<0.01). Although group IV had shorter postoperative eye-opening and extubation times compared with the other groups (P<0.05), it also had higher restlessness and pain scores (P<0.01). The combined use of sufentanil and remifentanil stabilizes perioperative hemodynamics and reduces stress hormone levels.

## Introduction

Stress reactions are strong and hemodynamic changes are great in elderly patients during the perioperative period. Therefore, issues concerning anesthetic methods and regulation of the stress reactions remain challenging and a focus of discussion in perioperative management for the elderly. Since remifentanil has an instant effect and rapid elimination that are not influenced by age or the functions of the liver and kidneys, and does not result in delayed recovery or respiratory depression after a continuous infusion, it has been extensively used in target-controlled infusion (TCI) for elderly patients (1–3). However, remifentanil readily causes postoperative acute pain which induces restlessness and stress reactions during recovery (4–6). This markedly increases the incidence of cerebrovascular accidents in the elderly. Postoperative acute pain following remifentanil use is correlated with the ultra-short-acting unique pharmacokinetics of remifentanil, which makes the pain more frequent and severe than those after the use of any other opioid (6) and is associated with opioid-induced hyperalgesia (OIH) (7–9).

Sufentanil is the most effective long-acting opioid receptor agonist discovered thus far; it has a high selectivity for the μ_1_ receptor but a low affinity for the δ receptor (10,11). Furthermore, sufentanil seldom leads to acute pain and OIH (4,5). The combined use of sufentanil with remifentanil during anesthesia effectively prevents acute pain during the recovery period following remifentanil use and may inhibit cardiovascular reactions during the extubation period (12). A preoperative epidural injection of 50 μg sufentanil significantly decreases the analgesic requirement at 6–12 h after abdominal surgery, reduces the secretion of adrenocorticotropic hormone (ACTH) and cortisol, and exerts a preemptive analgesic effect (13).

Despite this, the optimal schedule and doses of combined sufentanil and remifentanil for anesthesia of the elderly remain to be explored. Therefore, in the current study, propofol combined with different doses of sufentanil and remifentanil was administered to elderly patients by TCI. The effect of the combination on perioperative stress reactions and its analgesic effect were observed. The recommended respective doses of sufentanil and remifentanil and the combined medication method for anesthesia induction and maintenance were also explored.

## Patients and methods

### Subjects and grouping

A total of 80 elderly patients of American Society of Anesthesiology (ASA) I–II status admitted for elective abdominal surgery were recruited. Among the patients, 37 were subjected to gastrointestinal surgery, 28 to biliary surgery, 9 to urinary surgery and 6 to gynecological surgery. The patients ranged in age from 60 to 71 years and in weight from 46 to 78 kg that fluctuated within 20% of the standard weight or Body Mass Index (BMI). The surgical procedures lasted between 90 to 160 min. The subjects were randomly enrolled in groups 1, 2, 3 or 4 in a 1:1:1:1 ratio. Each subject was assigned a randomization number in ascending order and treated with the corresponding medication until the enrolled subjects in each site reached the scheduled number. All patients were free from airway difficulty, hearing disorder, a history of neuropsychiatric disorders, and a history of propofol or opioid allergy. This study was conducted in accordance with the Declaration of Helsinki and with approval from the Ethics Committee of The Provincial Hospital Affiliated to Anhui Medical University (Hefei, China). Written informed consent was obtained from all participants.

The patients were divided into four groups with 20 in each, according to different target concentrations of remifentanil and sufentanil. Group I received 4 ng/ml remifentanil and 0.2 ng/ml sufentanil for anesthesia induction and maintenance, group II received 3 ng/ml remifentanil and 0.3 ng/ml sufentanil, group III was subjected to 2 ng/ml remifentanil and 0.5 ng/ml sufentanil for anesthesia induction and post-intubation 3 ng/ml remifentanil and 0.2 ng/ml sufentanil for anesthesia maintenance, and group IV received 5 ng/ml remifentanil for anesthesia induction and then 4 ng/ml remifentanil for maintenance after intubation.

### Treatment methods

After venous opening, the electrocardiogram (ECG) and oxygen saturation (SpO_2_) were monitored, and invasive blood pressure was monitored after radial arterial cannulation. An Aspect A-2000XP EEG bispectral index (BIS) monitor (BIS™, Covidien, San Jose, CA, USA) was used for BIS continuous monitoring. A CP-600TCI injection pump (Beijing Slgo Medical Technology Co., Ltd., Beijing, China) was used for the respective injections of the Marsh, Minto and Bovill pharmacokinetic parameters of propofol (Batch No.: GL786; AstraZeneca, Caponago, Italy), remifentanil (Batch No.: 090512; Yichang Humanwell Pharmaceutical Co., Ltd., Yichang, China) and sufentanil (Batch No.: 091205; Yichang Humanwell Pharmaceutical Co., Ltd.).

### Anesthesia induction

Midazolam (0.05 mg/kg) was intravenously injected and then TCI with propofol at a target concentration of 4 μg/ml combined with remifentanil and sufentanil (the target concentrations were in line with those described above) was performed. When the BIS of the patient fell below 60, consciousness was lost. In addition, when the levels of the drugs reached the set target plasma concentrations, 0.9 mg/kg rocuronium bromide was intravenously injected to enable tracheal cannulation to be performed. An end-tidal partial pressure of carbon dioxide between 30 and 40 mmHg was maintained by mechanical ventilation.

### Anesthesia maintenance

The target concentration of propofol was reduced to 2 μg/ml following cannulation, while those of remifentanil and sufentanil were not adjusted. An intermittent intravenous injection of vecuronium bromide (50–80 μg/kg/h) was administered for Skelaxin maintenance.

For all patients, sufentanil was withdrawn prior to abdominal closure, and propofol and remifentanil were withdrawn at the time of skin suturing.

### Hemodynamics

The numbers of control due to severe hemodynamic fluctuation were recorded. When the contractive pressure decreased to <90 mmHg, or mean arterial pressure (MAP) was <60 mmHg for >1 min, ephedrine at 5 mg was administered. When the heart rate (HR) fell to <50 bpm for >1 min, atropine between 0.25 and 0.5 mg was administered. When the contractive pressure rose to >160 mmHg, diastolic pressure to >100 mmHg, or HR to >120 bpm for >1 min, 1 μg/kg remifentanil, 5 mg urapidil, or 25 mg esmolol was intravenously injected accordingly. During the perioperative period, patients received infusion with a 2:1 crystal/colloid ratio (6–8 ml/kg/h). Those with blood loss >20% were excluded from this study. Patients in all the groups breathed spontaneously during the extubation period. SpO_2_ was closely monitored. When SpO_2_ decreased to <90%, oxygen was supplied using a mask.

### Enzyme-linked immunosorbent assay (ELISA)

The HR, MAP, SpO_2_ and BIS values were recorded prior to anesthesia induction (T_0_), instantly before intubation (T_1_), 1 and 5 min after the intubation (T_2_ and T_3_), at the time of skin cutting (T_4_) and abdomen entry (T_5_), 30 min after entry (T_6_), before extubation (T_7_), and 1 and 5 min after the extubation (T_8_ and T_9_). Venous blood was extracted from 10 randomly selected patients from each group at T_0_, T_2_, T_5_, T_8_ and 3 h after the surgery (T_10_). The blood samples were placed into pre-chilled heparin tubes and then centrifuged for plasma isolation. The obtained plasma samples were cryopreserved for concentration determination of plasma catecholamines (NE and E) and angiotensin II (Ang II) using ELISA (the kit was supplied by Shanghai Senxiong Biotech Industry Co., Ltd., Shanghai, China).

### Postoperative scoring

The postoperative eye-opening and extubation times after drug withdrawal, restlessness (RS) and alertness/sedation scores (OAA/S) at 10 min after extubation, and pain visual analog score (VAS) at 3 h after surgery were recorded. The RS scoring criteria included: 0 for quiet and cooperation, 1 for limb movements stimulated by sputum sucking, 2 for struggles by no stimulation that, however, do not physical restraint, and 3 for intense struggles that require physical restraint. The OAA/S scoring criteria were as follows: 5 points for a fast response to name calling in a normal voice and complete consciousness; 4 for a slow response to name calling in a normal voice and a slow speech rate; 3 for a response to name calling only when a loud voice was used or the calling was repeated, slurred speech and glassy eyes; 2 for a response when nudging or patting was performed and incognizable speech; and 1 for no response when nudging or patting was performed, and lethargy.

### Statistical analysis

All data were analyzed using SPSS 11.5 software (SPSS, Inc., Chicago, IL, USA) and measurement data were presented as mean ± SD. One-way ANOVA was used for comparisons within and between groups, and the χ^2^ test was performed to compare enumeration data. P<0.05 was considered to indicate a statistically significant result.

## Results

### ELISA

Significant differences were not observed for age, weight, gender ratio or operating time among the four groups (P>0.05). In each group, the BIS decreased rapidly during anesthesia induction, stayed below 60 between T_1_ and T_6_, and then rose rapidly above 60 between T_7_ and T_9_. No significant difference was observed among the groups (P>0.05).

In each group, HR and MAP decreased at T_1_ (P<0.01) and increased at T_2_ compared with the values at T_0_. At T_2_, the MAP in group IV was higher than that in group II, and the HR and MAP of group IV were higher than those in group III (P<0.05). At T_8_ and T_9_, the HR and MAP in group IV were higher than those in any other group (P<0.05). At T_9_, the SpO_2_ levels in all the groups were lower than those at T_0_ (P<0.01); the level in group II was lower than that in group IV (P<0.05). The results are summarized in Table I.

Compared with the NE, N and Ang II values at T_0_, those at T_2_, T_5_ and T_8_ increased in each group (P<0.01). The values of these hormones in group IV at T_10_ were significantly higher than those in any other group (P<0.05 or P<0.01), the values at T_2_ in group IV were higher than those in group III (P<0.05). The results are summarized in Table II.

### Hemodynamics and postoperative scores

The intraoperative control number in group IV was significantly higher than that in any other group (P<0.01). The postoperative eye-opening and extubation times in this group after drug withdrawal were noticeably shorter than that in any other group (P<0.05 or P<0.01) but with higher post-extubation RS and VAS scores (P<0.01). Compared with group IV, group II showed prolonged eye-opening and extubation times (P<0.01). These results are summarized in Table III.

## Discussion

A combined use of opioids may reduce the respective doses of the drugs, as well as adverse reactions induced by their single use (12,14). Remifentanil is a type of opioid that is primarily metabolized and degraded by a non-specific esterase in blood plasma and tissues, while sufentanil is another type of opioid which is metabolized by the liver and kidneys and therefore has a long action time. Since remifentanil and sufentanil have different metabolic pathways and processes, they do not interfere with each other in metabolism and elimination when used in combination. The present study shows that the required target concentration of remifentanil in combination with sufentanil was significantly lower than that of remifentanil alone, when used in combination with propofol for anesthesia, which suggests that combined use of remifentanil and sufentanil greatly reduces their respective doses. This dose reduction is likely due to the high affinity of sufentanil for the μ_1_ receptor (11), as well as the synergistic effect of the two drugs.

In elderly patients, the functional reserve of the primary organs significantly decrease. A single intravenous injection easily induces a large drug level fluctuation in the blood which may lead to noticeable cardiovascular adverse reactions. By contrast, TCI may achieve a more accurate and stable blood drug level benefiting patient recovery (15). Remifentanil and sufentanil are applicable in TCI for the elderly (16,17). The median effective concentration (EC50) of remifentanil to inhibit cardiovascular responses at the time of tracheal cannulation and skin cutting are 5.0 and 2.1 ng/ml, respectively, when used in combination with propofol for anesthesia (18). Sufentanil at a plasma concentration of 0.4±0.2 ng/ml achieves favorable sedative and analgesic effects, as well as a low respiratory depression rate (19). Sufentanil at an effective concentration of 0.2 ng/ml combined with 4 μg/ml propofol for intraoperative anesthesia maintenance is the optimal anesthetic method which maintains intraoperative hemodynamic stability and greatly promotes patient postoperative recovery (20). Therefore, four different dose combinations of remifentanil and sufentanil were designed in this study. The results show that group IV, followed by group I, had the most noticeable hemodynamic fluctuation amplitude and the largest number of control due to severe hemodynamic fluctuation. Sufentanil has a dosage-dependent circulation inhibiting effect, and therefore, a small dosage should be used for elderly patients (21). Furthermore, this study shows that although the circulation in group II during surgery and at the time of extubation following surgery was stable, patient eye-opening and extubation times were prolonged, and SpO_2_ levels were low following extubation. These findings suggest that a large intraoperative maintenance dosage of sufentanil may induce the risk of postoperative respiratory depression.

Compared with a small dose, a large dose of remifentanil increases postoperative sensitivity to pain by 50% and increases the usage amounts of opioid analgesics by 85% (8). Although no objective method has been observed to directly identify whether it is OIH or tachyphylaxis that results in the increased use of postoperative analgesics, it is believed that opioid-mediated deallergization (resistance) and hypersensitization (hyperalgesia) may have the same pathogenesis (22,23). Opioids, apart from coupling with inhibitory G protein and producing an analgesic effect, also couple with excitatory G protein to activate the internal damage-promoting mechanism, thereby increasing the sensitivity of an organism to pain (24). Numerous studies have proved that remifentanil binds with the μ receptor to produce an analgesic effect, however, the effect is induced by the δ receptor to activate the NMDA receptor through the joint action of the μ and δ receptors to result in hyperalgesia (9,25). A recent study has shown that the prevention of the interaction between the spinal intramedullary δ and μ opioid receptors not only increases the analgesic effect of morphine but also decreases tolerance to morphine (26). Sufentanil has a binding affinity at the [^3^H]-DADL-labeled δ-binding site which is 100-fold lower than that at the μ-binding site (10). Opioids produce an analgesic effect primarily by exciting the μ_1_ receptor, whereas μ2 receptor excitation is principally responsible for adverse reactions, including respiratory depression and addiction. Sufentanil has high selectivity for the μ_1_ receptor. It has the most powerful analgesic effect among the fentanyl family and its analgesic effect is long-lasting. The therapeutic index of sufentanil (determined using the rat tail flicking method) is 25211 which is much higher than those of fentanyl (277) and morphine (69.5) (18). Its high selectivity for the μ_1_ receptor and low affinity for the δ receptor determine that it has a lower OIH effect than remifentanil. This finding provides a theoretical basis for the use of sufentanil to inhibit remifentanil-induced hyperalgesia. This study also demonstrated that the combined use of sufentanil and remifentanil with varying doses for anesthesia induction and maintenance markedly reduced postoperative early pain compared with a single use of remifentanil, particularly between 2 and 3 ng/ml remifentanil combined with a high dosage of sufentanil (0.5 ng/ml) for anesthesia induction and with a low dosage of sufentanil (0.2 ng/ml) for anesthesia maintenance (as in group III). However, whether such an effect is correlated with the preemptive analgesic or remaining effect of sufentanil remains to be explored. This study shows that the RS and OAA/S scores following extubation and VAS score at 3 h after surgery in group III were all significantly lower than those in group IV. Although the patients in group IV had short postoperative eye-opening and extubation times, they presented marked post-extubation restlessness and pain, as well as strong perioperative stress reactions.

Various negative perioperative incentives lead to a series of neuroendocrine responses, particularly sympathetic nerve excitation and pituitary ACTH hypersecretion, to negatively influence the organism. Plasma catecholamine concentration is a major index of stress reaction (27) and stress stimulation may increase the release of plasma catecholamines *in vivo* in a few seconds. Ang II is the most important bioactive substance of the renin-angiotensin system which excites the central AT1 receptor to cause sympathetic nerve excitation and to increase blood pressure (28). In the current study, the concentrations of plasma NE, N and Ang II in all the groups greatly increased at the time of tracheal cannulation, extubation and abdomen entry; group IV presented the most noticeable increases in these indices, which remained at high levels even after surgery, whereas group III had the lowest levels at the time of tracheal cannulation. These findings suggest that although a BIS <60 is not able to inhibit stress reactions in elderly patients completely, sufentanil has a more powerful inhibitory effect on these reactions than remifentanil.

To summarize, the combined use of sufentanil and remifentanil of varying doses markedly reduces the postoperative early acute pain caused by remifentanil. A dose of 2 ng/ml remifentanil + 0.5 ng/ml sufentanil for anesthesia induction and 3 ng/ml remifentanil + 0.2 ng/ml sufentanil post-intubation for anesthesia maintenance for elderly patients stabilized peri-operative hemodynamics, decreased stress hormone levels and reduced the respective dosages of remifentanil and sufentanil. In addition, this method leads to only slight adverse reactions.

## Figures and Tables

**Table I.** Comparisons of HR, MAP, SpO_2_ and BIS among the four groups at different time points (n=20, mean ± SD).

Index	Group	T_0_	T_1_	T_2_	T_3_	T_4_	T_5_	T_6_	T_7_	T_8_	T_9_
HR (bpm)	I	81±16	70±11^b^	90±16^b^	78±13	84±14	85±15	79±12	88±13^a^	92±16^b,^^c^	79±16^c^
	II	80±17	71±12^b^	87±15^a^	76±12	81±16	81±14	76±15	84±16^c^	88±15^a,c^	76±13^c^
	III	79±15	69±11^b^	84±17^c^	75±12	81±15	82±16	78±14	83±15^c^	87±14^a,c^	77±14^c^
	IV	81±15	70±10^b^	96±17^b^	80±14	86±13	87±13	83±15	95±16^b^	103±19^b^	89±15^a^
MAP (mmHg)	I	89±10	74±8^b^	95±12^a^	82±7^a^	86±8	87±9	82±8	88±9	92±10^a,c^	85±8^c^
	II	90±9	75±9^b^	89±11^c^	80±6^a^	85±7	86±8	80±7	84±10	89±11^c^	83±7^c^
	III	89±9	76±10^b^	86±10^c^	79±7^a^	83±8	85±9	83±8	85±9	90±11^c^	84±8^c^
	IV	88±11	73±7^b^	99±13^b^	86±9	89±10	91±11	85±10	90±9	101±11^b^	92±10^a^
SpO_2_ (%)	I	98.3±0.7	99±0	99±0	99±0	99±0	99±0	99±0	99±0	99±0	95.9±1.8^b^
	II	98.4±0.8	99±0	99±0	99±0	99±0	99±0	99±0	99±0	99±0	94.6±2.2^b,c^
	III	98.3±0.8	99±0	99±0	99±0	99±0	99±0	99±0	99±0	99±0	95.8±1.7^b^
	IV	98.2±0.8	99±0	99±0	99±0	99±0	99±0	99±0	99±0	99±0	96.2±1.8^b^
BIS	I	96±2	49±7	50±8	48±6	47±8	49±8	49±7	87±8	89±5	89±3
	II	95±3	46±7	47±7	47±8	48±6	49±7	48±8	88±7	90±4	90±5
	III	96±3	48±6	48±8	50±5	49±7	48±6	47±6	89±8	91±4	91±4
	IV	96±2	47±8	49±7	48±8	47±7	49±7	48±7	88±9	91±6	92±3

a P<0.05 and

b P<0.01 compared with T_0_;

c P<0.05 compared with group IV. HR, heart rate; MAP, mean arterial pressure; SpO_2_, oxygen saturation; BIS, bispectral index.

**Table II.** Comparisons of plasma NE, E and Ang II among the four groups at different time points (n=10, mean ± SD).

Index	Group	T_0_	T_2_	T_5_	T_8_	T_10_
NE (pg/ml)	I	317±69	402±89^b^	365±76^b^	377±73^b^	335±63
	II	326±76	375±84^b^	358±72^b^	369±64^b^	317±60^c^
	III	316±62	359±73^b,c^	349±69^b^	365±65^b^	320±62^c^
	IV	321±80	417±91^b^	381±85^b^	403±89^b^	365±68^b^
E (pg/ml)	I	207±38	291±53^b^	262±48^b^	276±49^b^	220±48
	II	204±43	279±56^b^	253±45^b^	261±43^b^	208±42^c^
	III	212±32	263±47^b,c^	246±42^b^	258±46^b^	211±47^c^
	IV	201±40	302±58^b^	270±53^b^	287±56^b^	247±53^b^
Ang II (pg/ml)	I	41±11	59±14^b^	49±11^b^	53±13^b^	40±11
	II	36±9	55±12^b^	47±13^b^	49±11^b^	37±9^c^
	III	38±8	50±10^b,c^	46±11^b^	48±9^b^	38±10^c^
	IV	39±10	64±17^b^	53±14^b^	56±14^b^	46±13^a^

a P<0.05 and

b P<0.01 compared with T_0_;

c P<0.05 compared with group IV. NE, norephinephrine; E, epinephrine; Ang II, Angiotensin II.

**Table III.** Comparisons of the intraoperative circulation fluctuation control and postoperative conditions among the four groups (n=20, mean ± SD).

Group	Control number	Eye-opening time (min)	Extubation time (min)	RS	OAA/S	VAS at 3 h after surgery
I	2.5±1.5^b^	8.9±3.6^a^	12.4±4.3^a^	0.6±0.6^b^	4.2±1.0	3.7±2.6
II	1.9±1.0^b^	10.3±3.5^b^	17.5±4.2^b^	0.4±0.5^b^	4.1±1.0	2.3±1.5^b^
III	2.1±1.1^b^	9.3±3.8^a^	12.9±4.1^a^	0.5±0.6^b^	4.2±1.0	2.5±1.8^b^
IV	4.2±1.8	6.4±2.8	9.5±3.8	1.5±1.1	4.5±0.7	5.7±3.3

a P<0.05 and

b P<0.01 compared with group IV. RS, restlessness at 10 min after extubation; OAA/S, alertness/sedation scores at 10 min after extubation; VAS, pain visual analog score.

## References

[1] Sieber FE, Pauldine R, Miller RD (2010). Geriatric anesthesia. Miller’s anesthesia.

[2] Park SH, Jeong ST, Tak YJ, Kim CS, Kim ST (2010). A comparison of the hemodynamic changes and propofolinduced pain at two different doses of remifentanil in elderly patients. Korean J Anesthesiol.

[3] Ouattara A, Boccara G, Lemaire S (2003). Target-controlled infusion of propofol and remifentanil in cardiac anaesthesia: influence of age on predicted effect-site concentrations. Br J Anaesth.

[4] Derrode N, Lebrun F, Levron JC, Chauvin M, Debaene B (2003). Influence of peroperative opioid on postoperative pain after major abdominal surgery: sufentanil TCI versus remifentanil TCI. A randomized, controlled study. Br J Anaesth.

[5] Motamed C, Merle JC, Yakhou L (2006). Postoperative pain scores and analgesic requirements after thyroid surgery: comparison of three intraoperative opioid regimens. Int J Med Sci.

[6] Michelsen LG, Hug CC (1996). The pharmacokinetics of remifentanil. J Clin Anesth.

[7] Lee C, Song YK, Lee JH, Ha SM (2011). The effects of intraoperative adenosine infusion on acute opioid tolerance and opioid induced hyperalgesia induced by remifentanil in adult patients undergoing tonsillectomy. Korean J Pain.

[8] Guignard B, Bossard AE, Coste C (2000). Acute opioid tolerance: intraoperative remifentanil increases postoperative pain and morphine requirement. Anesthesiology.

[9] Gu X, Wu X, Liu Y, Cui S, Ma Z (2009). Tyrosine phosphorylation of the N-Methyl-D-Aspartate receptor 2B subunit in spinal cord contributes to remifentanil-induced postoperative hyperalgesia: the preventive effect of ketamine. Mol Pain.

[10] Leysen JE, Gommeren W, Niemegeers CJ (1983). [^3^H] Sufentanil, a superior ligand for mu-opiate receptors: binding properties and regional distribution in rat brain and spinal cord. Eur J Pharmacol.

[11] Niemegeers CJ, Schellekens KH, Van Bever WF, Janssen PA (1976). Sufentanil, a very potent and extremely safe intravenous morphine-like compound in mice, rats and dogs. Arzneimittelforschung.

[12] Cafiero T, Di Minno RM, Sivolella G, Di Iorio C (2004). Immediate postoperative pain management in patients undergoing major abdominal surgery after remifentanil-based anesthesia: sufentanil vs tramadol. Minerva Anestesiol.

[13] Akural EI, Salomäki TE, Tekay AH, Bloigu AH, Alahuhta SM (2002). Pre-emptive effect of epidural sufentanil in abdominal hysterectomy. Br J Anaesth.

[14] Carrer S, Bocchi A, Candini M, Donegà L, Tartari S (2007). Short term analgesia based sedation in the Intensive Care Unit: morphine vs remifentanil + morphine. Minerva Anestesiol.

[15] Nora FS (2008). Total intravenous anesthesia as a target-controlled infusion. An evolutive analysis. Rev Bras Anestesiol.

[16] Zhao Y, Zhang LP, Wu XM (2009). Clinical evaluation of target controlled infusion system for sufentanil administration. Chin Med J (Engl).

[17] Yang XY, Xu X, Wu XM (2011). A comparison of remifentanil versus sufentanil with target-controlled infusion in combined inhalation anesthesia for surgical patients: effects on hemodynamics and postoperative recovery. Zhonghua Yi Xue Za Zhi.

[18] Albertin A, Casati A, Federica L (2005). The effect-site concentration of remifontanil blunting cardiovascular responses to tracheal intubation and skin incision during bispectral index-guided prpofol anesthesia. Anesth Analg.

[19] Bourgoin A, Albanèse J, Léone M, Sampol-Manos E, Viviand X, Martin C (2005). Effects of sufentanil or ketamine administered in target-controlled infusion on the cerebral hemodynamics of severely brain-injured patients. Crit Care Med.

[20] Hentgen E, Houfani M, Billard V, Capron F, Ropars JM, Travagli JP (2002). Propofol-sufentanil anesthesia for thyroid surgery: optimal concentrations for hemodynamic and electroencephalogram stability, and recovery features. Anesth Analg.

[21] Habib AS, Parker JL, Maguire AM, Rowbotham DJ, Thompson JP (2002). Effects of remifentanil and alfentanil on the cardiovascular responses to induction of anaesthesia and tracheal intubation in the elderly. Br J Anaesth.

[22] Célèrier E, Laulin JP, Corcuff JB, Le Moal M, Simonnet G (2001). Progressive enhancement of delayed hyperalgesia induced by repeated heroin administration: a sensitization process. J Neurosci.

[23] Colvin LA, Fallon MT (2010). Opioid induced hyperalgesia: a clinical challenge. Br J Anaesth.

[24] Tsai RY, Tai YH, Tzeng JI, Cherng CH, Yeh CC, Wong CS (2009). Ultra-low dose naloxone restores the antinociceptive effect of morphine in pertussis toxin-treated rats by reversing the coupling of mu-opioid receptors from Gs-protein to coupling to Gi-protein. Neuroscience.

[25] Zhao M, Joo DT (2008). Enhancement of spinal N-methyl-D-aspartate receptor function by remifentanil action at delta-opioid receptors as a mechanism for acute opioid-induced hyperalgesia or tolerance. Anesthesiology.

[26] He SQ, Zhang ZN, Guan JS (2011). Facilitation of µ-opioid receptor activity by preventing δ-opioid receptor-mediated codegradation. Neuron.

[27] Breslow MJ (1992). The role of stress hormones in perioperative myocardial ischemia. Int Anesthesiol Clin.

[28] Hitomi H, Kiyomoto H, Nishiyama A (2007). Angiotensin II and oxidative stress. Curr Opin Cardiol.

